# BiVO_4_/Fe_3_O_4_@polydopamine superparticles for tumor multimodal imaging and synergistic therapy

**DOI:** 10.1186/s12951-021-00802-x

**Published:** 2021-03-29

**Authors:** Ze Wang, Guan Wang, Tingting Kang, Shuwei Liu, Lu Wang, Haoyang Zou, Yu Chong, Yi Liu

**Affiliations:** 1 State Key Laboratory of Supramolecular Structure and Materials, Jilin University, Changchun, 130012 People’s Republic of China; 2 Key Laboratory of Polymer Ecomaterials, Changchun Institute of Applied Chemistry, Chinese Academy of Sciences, Changchun, 130012 People’s Republic of China; 3 State Key Laboratory of Radiation Medicine and Protection, School for Radiological and Interdisciplinary Sciences (RAD-X), Collaborative Innovation Center of Radiation Medicine of Jiangsu Higher Education Institutions, Soochow University, Suzhou, 215123 People’s Republic of China; 4 Department of Gastroenterology, China-Japan Union Hospital, Jilin University, Changchun, 130033 People’s Republic of China; 5 Department of Oral Pathology, School and Hospital of Stomatology, Jilin University, Changchun, 130021 People’s Republic of China

**Keywords:** Fe_3_O_4_ nanoparticles, BiVO_4_ nanoparticles, Superparticles, Multimodal imaging, Synergy therapy

## Abstract

**Background:**

Despite tremendous progress has been achieved in tumor theranostic over the past decade, accurate identification and complete eradication of tumor cells remain a great challenge owing to the limitation of single imaging modality and therapeutic strategy.

**Results:**

Herein, we successfully design and construct BiVO_4_/Fe_3_O_4_@polydopamine (PDA) superparticles (SPs) for computed tomography (CT)/photoacoustic (PA)/magnetic resonance (MR) multimodal imaging and radiotherapy (RT)/photothermal therapy (PTT) synergistic therapy toward oral epithelial carcinoma. On the one hand, BiVO_4_ NPs endow BiVO_4_/Fe_3_O_4_@PDA SPs with impressive X-ray absorption capability due to the high X-ray attenuation coefficient of Bi, which is beneficial for their utilization as radiosensitizers for CT imaging and RT. On the other hand, Fe_3_O_4_ NPs impart BiVO_4_/Fe_3_O_4_@PDA SPs with the superparamagnetic property as a T_2_-weighted contrast agent for MR imaging. Importantly, the aggregation of Fe_3_O_4_ NPs in SPs and the presence of PDA shell greatly improve the photothermal conversion capability of SPs, making BiVO_4_/Fe_3_O_4_@PDA SPs as an ideal photothermal transducer for PA imaging and PTT. By integrating advantages of various imaging modalities (CT/PA/MR) and therapeutic strategies (RT/PTT), our BiVO_4_/Fe_3_O_4_@PDA SPs exhibit the sensitive multimodal imaging feature and superior synergistic therapeutic efficacy on tumors.

**Conclusions:**

Since there are many kinds of building blocks with unique properties appropriating for self-assembly, our work may largely enrich the library of nanomateirals for tumor diagnosis and treatment. 
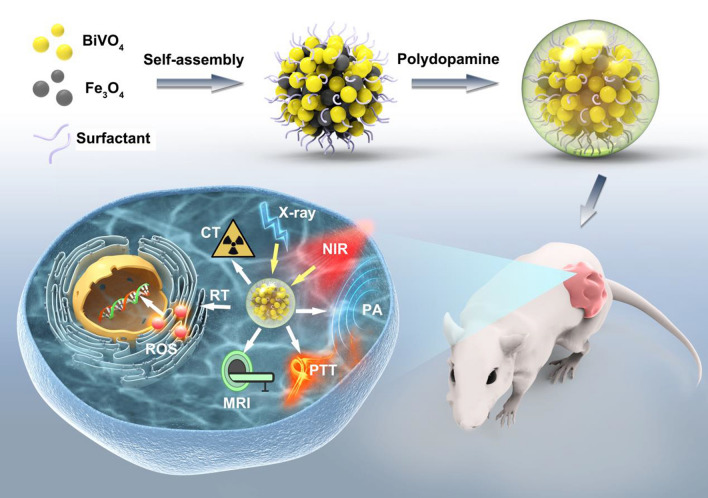

## Background

Radiotherapy (RT) is one of the most widely used clinical strategies for cancer treatment [1–4]. More than 50% of cancer patients suffering from solid tumors are under treatment of RT [5]. Benefiting from the high intensity ionizing radiations (such as electrons, protons, and photons), RT can directly introduce deoxyribonucleic acid (DNA) double-strand breaks or yield a large number of cytotoxic reactive oxygen species (ROS) to trigger the apoptosis or necrosis of irradiated cancer cells [6–9]. However, the therapeutic efficacy of conventional RT is still limited by the insufficient radiation energy deposition on tumor tissues, as well as the serious toxic effects on normal surrounding healthy tissues [10, 11]. Along with the development of nanotechnology and materials science, nanomaterials containing high-Z elements are exploited as radiosensitizers due to their high radiation energy deposition, thereby amplify the radiation-induced damage on tumor tissues, accompanied by the alleviation of the relevant side effects [12–15]. On the other hand, deriving from the abnormal blood vasculature, hypoxia is considered as the hostile feature of the tumor microenvironment (TME) to cause irreversible tumor metastasis and RT resistance [16–18]. As a novel therapeutic approach, photothermal therapy (PTT) is an efficient way to overcome this problem [19–21]. Under NIR light irradiation, photothermal agents not only generate regional hyperthermia to ablate cancer cells, but also promote the blood flow and oxygen pressure levels in tumor tissues, resulting in enhancing the tumor cell sensitivity to RT. Therefore, it is reasonable to believe that a superior synergistic therapeutic efficacy from RT and PTT will be achieved by taking advantages of radiosensitizers and photothermal agents simultaneously.

It is well known that imaging techniques play the pivotal role in clinical diagnosis and efficacy evaluation [22]. Although various imaging techniques have been rapidly developed for many years, information collected by a single imaging modality is usually limited and insufficient due to the intrinsic restrictions of each modality [23]. For example, computed tomography (CT) imaging is good at constructing 3D visualization with anatomical details, but suffering from the low resolution [24]. Magnetic resonance (MR) imaging can scan objects with high resolution, but at the expense of the time-consuming data acquisition process [25]. Photoacoustic (PA) imaging is capable of providing the fast real-time monitor and unveils information with high signal-to-noise ratio, but only appropriate to soft tissues [26]. Hence, integrating different imaging modalities into a single nanostructure may hold great potentials to achieve complementary information for tumor diagnosis precisely, accurately and efficiently. Because of the high X-ray absorption coefficient (5.74 cm^2^ g^− 1^ at 100 keV) and low cytotoxicity, bismuth (Bi)-based nanomaterials can be seen as an ideal radiosensitizer for CT imaging and RT [27]. Since most of previous Bi-based radiosensitizers focus on Bi_2_S_3_ and BiOX (X = Cl, Br, I), other Bi-based nanomaterials are still needed to be explored to enrich their candidates [28, 29]. In addition, due to the excellent superparamagnetic, Fe_3_O_4_ NPs have been approved by FDA for T_2_-weighted MR imaging [30]. Most recently, it is reported that Fe_3_O_4_ NP aggregates exhibit an enhanced photothermal conversion capability comparing to their individual NPs owning to the collective effect [31]. The as-prepared Fe_3_O_4_ NP aggregates are also verified to have the potential to be used as the photothermal agents for PA imaging and PTT. Thus, constructing nanostructures containing Bi-based nanomaterials and Fe_3_O_4_ NPs is of great significance in realizing the multimodal imaging and synergistic therapy.

Self-assembly, which mainly depend on the supramolecular interactions (including van der Waals (vdW) interaction, electrostatic interaction, dipole interaction, hydrogen bonding, hydrophobic interaction and π-π stacking interaction) between building blocks, has been widely used to construct assemblies with different morphologies and formations [32]. During self-assembly, the intrinsic physical and chemical properties of the building blocks are usually passed to the resulting assemblies entirely, which offer us a simple and flexible method to construct nanostructures with desired compositions and functions [33]. Herein, we successfully design and prepare BiVO_4_/Fe_3_O_4_@polydopamine (PDA) superparticles (SPs) for CT/PA/MR multimodal imaging and RT/PTT synergistic therapy. BiVO_4_ and Fe_3_O_4_ NPs with the average sizes of 7.12 and 5.49 nm are firstly prepared, followed by their subsequent self-assembly into BiVO_4_/Fe_3_O_4_ SPs via the oil-in-water microemulsion route. After that, the as-prepared BiVO_4_/Fe_3_O_4_ SPs are covered by PDA to further improve their photothermal conversion capability. At last, the imaging and therapy performances of BiVO_4_/Fe_3_O_4_@PDA SPs are evaluated via in vitro and in vivo experiments. The results clearly manifest that our BiVO_4_/Fe_3_O_4_@PDA SPs can be seen as the potential nanomedicine for tumor theranostic.

## Results and discussion

**Fig. 1:**
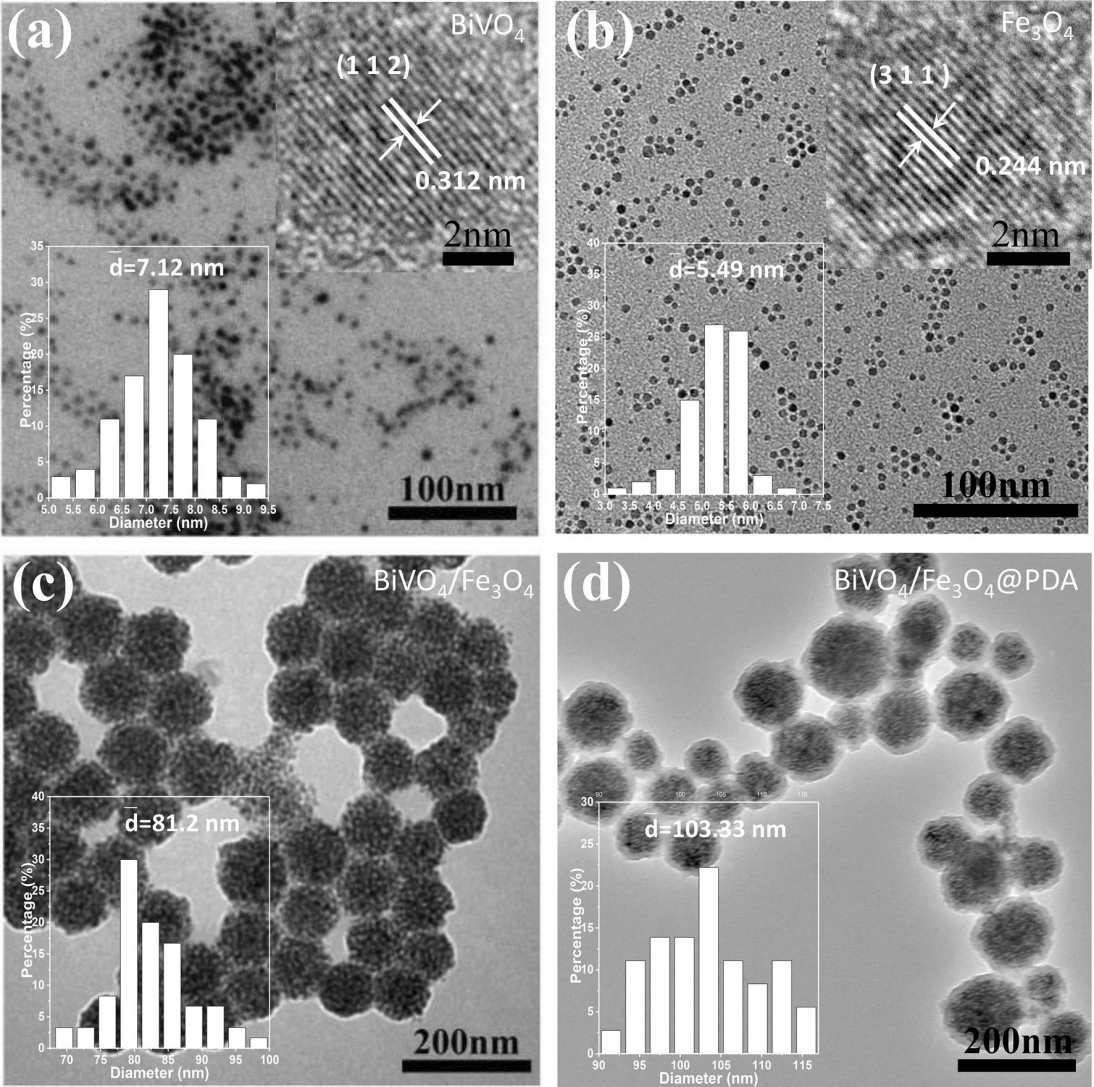
TEM and HRTEM characterizations of BiVO_4_ NPs, Fe_3_O_4_ NPs, BiVO_4_/Fe_3_O_4_ SPs and BiVO_4_/Fe_3_O_4_@PDA SPs. TEM images of (**a**) BiVO_4_ NPs, (**b**) Fe_3_O_4_ NPs, (**c**) BiVO_4_/Fe_3_O_4_ SPs and (**d**) BiVO_4_/Fe_3_O_4_@PDA SPs. Inset in (**a**) and (**b**): size distributions and HRTEM images of BiVO_4_ and Fe_3_O_4_ NPs. Inset in (**c**) and (**d**): size distributions of BiVO_4_/Fe_3_O_4_ SPs and BiVO_4_/Fe_3_O_4_@PDA SPs

BiVO_4_/Fe_3_O_4_@PDA SPs are constructed upon the self-assembly of BiVO_4_ and Fe_3_O_4_ NPs following by coating with the PDA shell. Typically, BiVO_4_ NPs are prepared through our previous two-phase method, and Fe_3_O_4_ NPs are prepared via the classical thermal decomposition method [34, 35]. Transmission electron microscopy (TEM) images in Fig. 1a and b show that both of BiVO_4_ and Fe_3_O_4_ NPs are monodispersed nanospheres with the average diameters around 7.12 and 5.49 nm, respectively. High-resolution TEM (HRTEM) images exhibit the lattice fringes with the interplanar spacings of 0.312 and 0.244 nm, corresponding to the (112) planes of monoclinic BiVO_4_ and the (311) planes of cubic Fe_3_O_4_. X-ray diffraction (XRD) patterns of BiVO_4_ and Fe_3_O_4_ NPs further identify the monoclinic crystal structure of BiVO_4_ NPs and the cubic crystal structure of Fe_3_O_4_ NPs (Additional file 1: Figure S1).

Then, oil-in-water microemulsion method is employed to construct BiVO_4_/Fe_3_O_4_ SPs using BiVO_4_ and Fe_3_O_4_ NPs as the building blocks while sodium dodecyl sulfate (SDS) as the surfactants [36]. The as-prepared SDS-capped BiVO_4_/Fe_3_O_4_ SPs are nanospheres with the average diameter of 81.20 nm (Fig. 1c). The element distributions of BiVO_4_/Fe_3_O_4_ SPs are characterized by energy-dispersive X-ray spectroscopy (EDS) elemental mapping (Additional file 1: Figure S2). Bi, V and Fe are uniformly distributed throughout the entire SPs, further demonstrating the assembled configuration of the as-prepared BiVO_4_/Fe_3_O_4_ SPs. Benefiting from the flexibility of the self-assembly technique, the size and composition of BiVO_4_/Fe_3_O_4_ SPs is tunable deliberately. For example, by increasing the toluene-to-water ratio from 1:5 to 2:5, the size of BiVO_4_/Fe_3_O_4_ SPs can be increased from 81.20 to 164.50 nm (Additional file 1: Figure S3). In the meantime, upon adjusting the feeding ratio between BiVO_4_ and Fe_3_O_4_ NPs during self-assembly, the molar ratio of Bi/Fe in the as-prepared BiVO_4_/Fe_3_O_4_ SPs is varied from 3.5:1 to 1.2:1. The corresponding products are designated as BiVO_4_/Fe_3_O_4_-1, BiVO_4_/Fe_3_O_4_-2 and BiVO_4_/Fe_3_O_4_-3 SPs (Additional file 1: Table S1).

At last, dopamine (DA) monomers are oxidized followed by spontaneous polymerization on the surface of BiVO_4_/Fe_3_O_4_ SPs under the alkaline condition [37]. The thickness of the PDA shell is positively correlated with the amount of DA, which will be increased from 10.00 to 80.00 nm when the concentration of DA increases from 0.3 to 0.8 mg/mL (Additional file 1: Figure S4). Given that nanomaterials larger than 120.00 nm can hardly enter into cells upon cellular phagocytosis, BiVO_4_/Fe_3_O_4_ SPs with the diameter around 80.00 nm is selected as the core and the PDA shell thickness is designed to be around 10.00 nm.

**Fig. 2:**
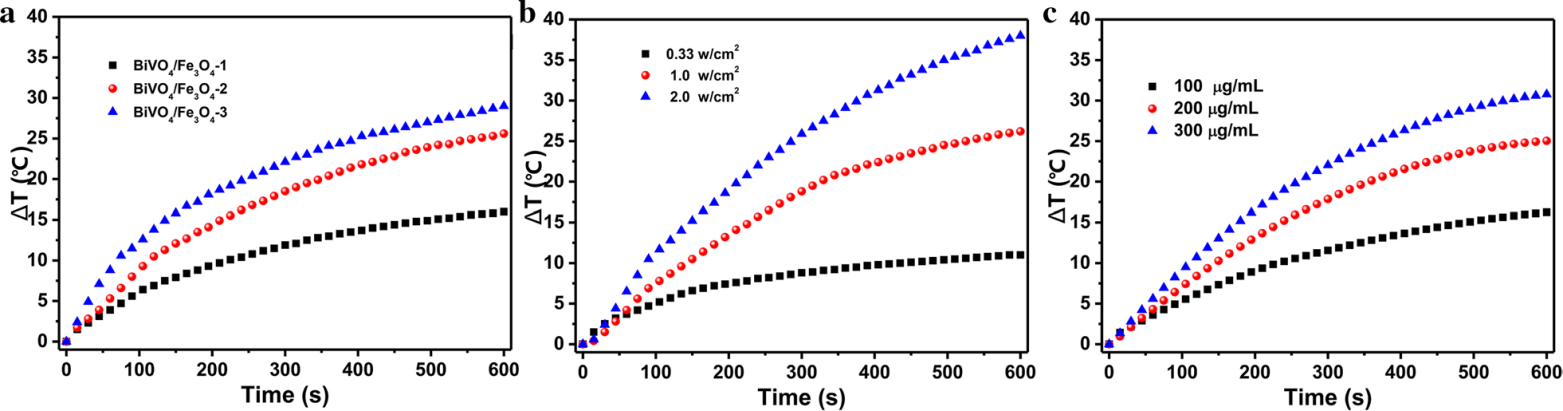
Photothermal conversion capability characterizations of BiVO_4_/Fe_3_O_4_@PDA SPs. Temperature increments vs. the proportions of Fe_3_O_4_ in BiVO_4_/Fe_3_O_4_@PDA SPs (200 µg/mL SPs; 1 W/cm^2^ irradiation) (**a**), the power densities of incident laser (200 µg/mL BiVO_4_/Fe_3_O_4_-2@PDA SPs) (**b**), the concentrations of SPs (BiVO_4_/Fe_3_O_4_-2@PDA SPs; 1 W/cm^2^ irradiation) (**c**). The molar ratios of BiVO_4_:Fe_3_O_4_ in SPs from BiVO_4_/Fe_3_O_4_-1 to BiVO_4_/Fe_3_O_4_-3 are 10.5:1, 5.4:1 and 3.6:1

Since the aggregation of Fe_3_O_4_ NPs and the presence of the PDA shell can remarkably increase the molar extinction coefficient of monodispersed Fe_3_O_4_ NPs (Additional file 1: Figure S5), leading to the enhancement in their photothermal conversion capability, the photothermal conversion capability of BiVO_4_/Fe_3_O_4_@PDA SPs suspension is evaluated under 808 nm irradiation. As shown in Fig. 2a, the photothermal conversion capability of BiVO_4_/Fe_3_O_4_@PDA SPs is enhanced by elevating the proportion of Fe_3_O_4_ in SPs. At the same time, the temperature of BiVO_4_/Fe_3_O_4_@PDA SPs aqueous solution rises rapidly by increasing the power density of the applied laser and the concentration of SPs (Fig. 2b and c). Under 1 W/cm^2^ irradiation for 10 min, the aqueous solution containing 200 µg/mL of BiVO_4_/Fe_3_O_4_@PDA SPs exhibits a noticeable temperature increment of 25 °C. Based on the reported model, the photothermal conversion efficiency of as-prepared BiVO_4_/Fe_3_O_4_@PDA SPs is estimated to be 33.42%, which is comparable to previous reports (Additional file 1: Figure S6) [31]. To balance the properties deriving from BiVO_4_ and Fe_3_O_4_, BiVO_4_/Fe_3_O_4_@PDA SPs with the Bi/Fe element ratio of 1.8/1 are selected for the following in vitro and in vivo experiments.

Prior to assessing the imaging performance of BiVO_4_/Fe_3_O_4_@PDA SPs, their cytotoxicity is evaluated via standard Cell Counting Kit 8 (CCK-8) assay. After incubation with BiVO_4_/Fe_3_O_4_@PDA SPs at different concentrations for 24 h, the cell viability of oral epithelial carcinoma (KB) cells is higher than 80% even at a high concentration of 300 µg/mL, which strongly manifest the negligible cytotoxicity of BiVO_4_/Fe_3_O_4_@PDA SPs (Additional file 1: Figure S7). The colloidal stability of BiVO_4_/Fe_3_O_4_@PDA SPs is tested as well. After storage in water, saline, cell culture or serum-containing cell culture for 24 h, BiVO_4_/Fe_3_O_4_@PDA SPs are well dispersed without any visible coagulation (Additional file 1: Figure S8). The low toxicity plus the high colloidal stability provide a powerful guarantee for the utilization of BiVO_4_/Fe_3_O_4_@PDA SPs in tumor theranostic.

**Fig. 3:**
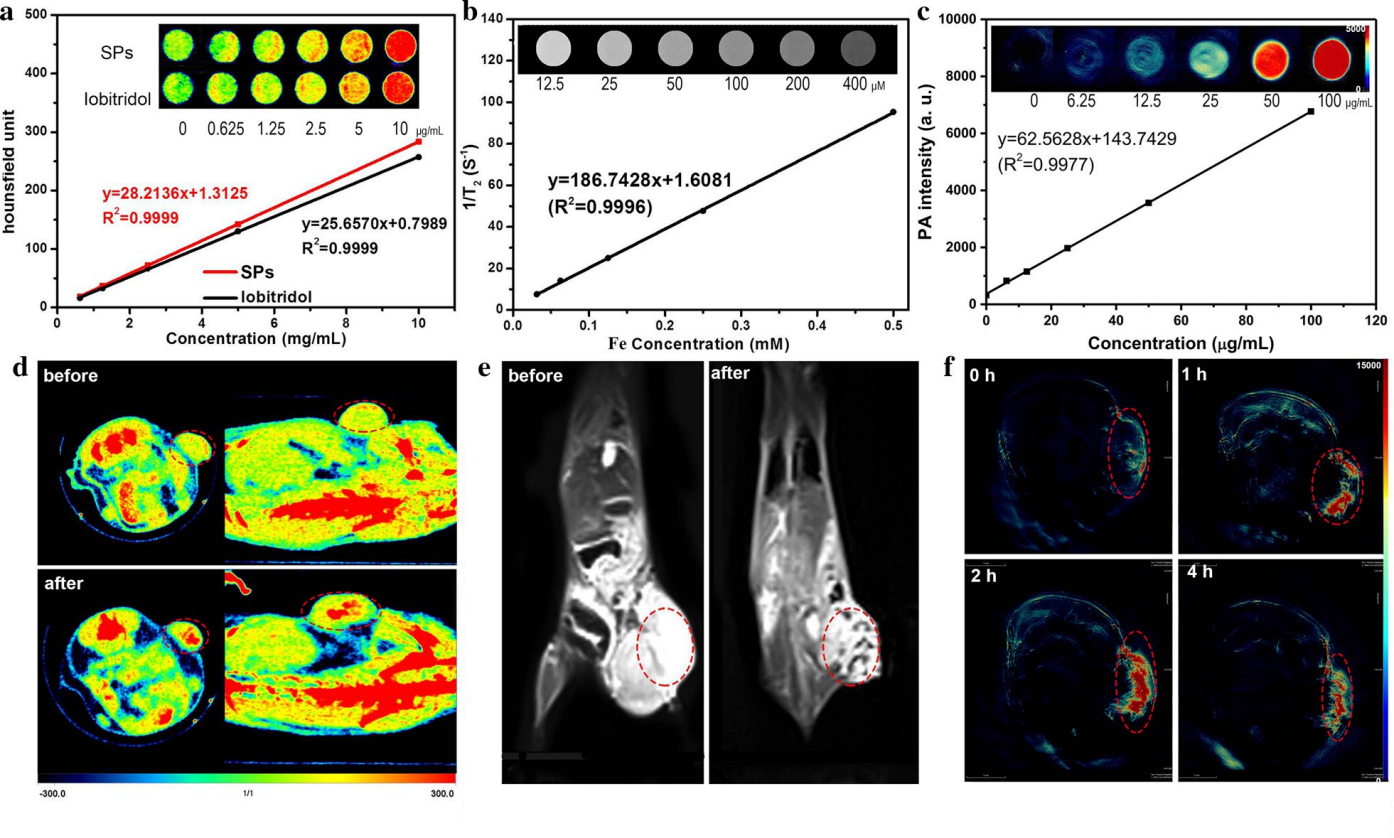
CT/MRI/PA imaging properties of BiVO_4_/Fe_3_O_4_@PDA SPs in vitro and in vivo. **a** In vitro CT images and HU values of BiVO_4_/Fe_3_O_4_@PDA SPs and iobitridol solution at different concentrations. **b** In vitro T_2_-weighted MR images and T_2_ relaxation rates of BiVO_4_/Fe_3_O_4_@PDA SPs at different concentrations. **c** In vitro PA images and PA values of BiVO_4_/Fe_3_O_4_@PDA SPs at different concentrations. In vivo CT (**d**), MR (**e**) and PA (**f**) images of mice bearing KB tumors obtained before and after intratumoral injection of BiVO_4_/Fe_3_O_4_@PDA SPs

Subsequently, the in vitro imaging performances of BiVO_4_/Fe_3_O_4_@PDA SPs are exhibited in Fig. 3a–c. Owning to the high X-ray attenuation coefficient of Bi, the CT signal intensities of BiVO_4_/Fe_3_O_4_@PDA SPs increase linearly and sharply with their concentrations. The Hounsfield units (HU) value of BiVO_4_/Fe_3_O_4_@PDA SPs is calculated to be 28.2136 HU mL mg^− 1^, which is comparable to the clinically used CT contrast agent iobitridol (25.6570 HU mL mg^− 1^) (Fig. 3a). Further increasing the proportion of BiVO_4_ in SPs can improve the CT imaging performance of BiVO_4_/Fe_3_O_4_@PDA SPs undoubtedly, but may lose their PA and MR imaging performances as the price. In addition, BiVO_4_/Fe_3_O_4_@PDA SPs are also anticipated to be the MR imaging contrast agents owing to the superparamagnetic property of Fe_3_O_4_ NPs. Their MR imaging contrast is enhanced in a concentration-dependent manner, and the r_2_ value is estimated to be 186 mM^− 1 ^s^− 1^, which is higher than current commercial MR contrasts, such as Resovist (143 mM^− 1 ^s^− 1^) and Feridex (93 mM^− 1 ^s^− 1^) (Fig. 3b). Furthermore, benefiting from the excellent photothermal conversion capability, there is a good linear relationship between the concentration of BiVO_4_/Fe_3_O_4_@PDA SPs and their PA signal under NIR irradiation, suggesting their great potentials as the PA contrast agents (Fig. 3c). Then, in order to prove the concept that our BiVO_4_/Fe_3_O_4_@PDA SPs have the potential to be used for imaging in vivo, multimode CT/MR/PA imaging properties of BiVO_4_/Fe_3_O_4_@PDA SPs are explored on the subcutaneous tumor model. As shown in Fig. 3d, the tumor tissue will possess the enhanced CT imaging signal after the intratumoral injection of BiVO_4_/Fe_3_O_4_@PDA SPs. In contrast, only normal bone structures can be observed without the injection of BiVO_4_/Fe_3_O_4_@PDA SPs. Meanwhile, the mouse treated by BiVO_4_/Fe_3_O_4_@PDA SPs displays a clear T_2_-weighted MR imaging in the tumor region comparing to the region without the SP injection (Fig. 3e). As for PA imaging, the PA signal of tumor is notably enhanced after intratumoral injection of BiVO_4_/Fe_3_O_4_@PDA SPs. As a comparison, only extremely weak PA signal arising from the tumor blood can be detected in the tumor site without the injection of BiVO_4_/Fe_3_O_4_@PDA SPs (Fig. 3f). The results above suggest the great potentials of BiVO_4_/Fe_3_O_4_@PDA SPs in multimodal imaging, which could combine advantages of each technique to provide complementary information for accurate diagnosis.

**Fig. 4:**
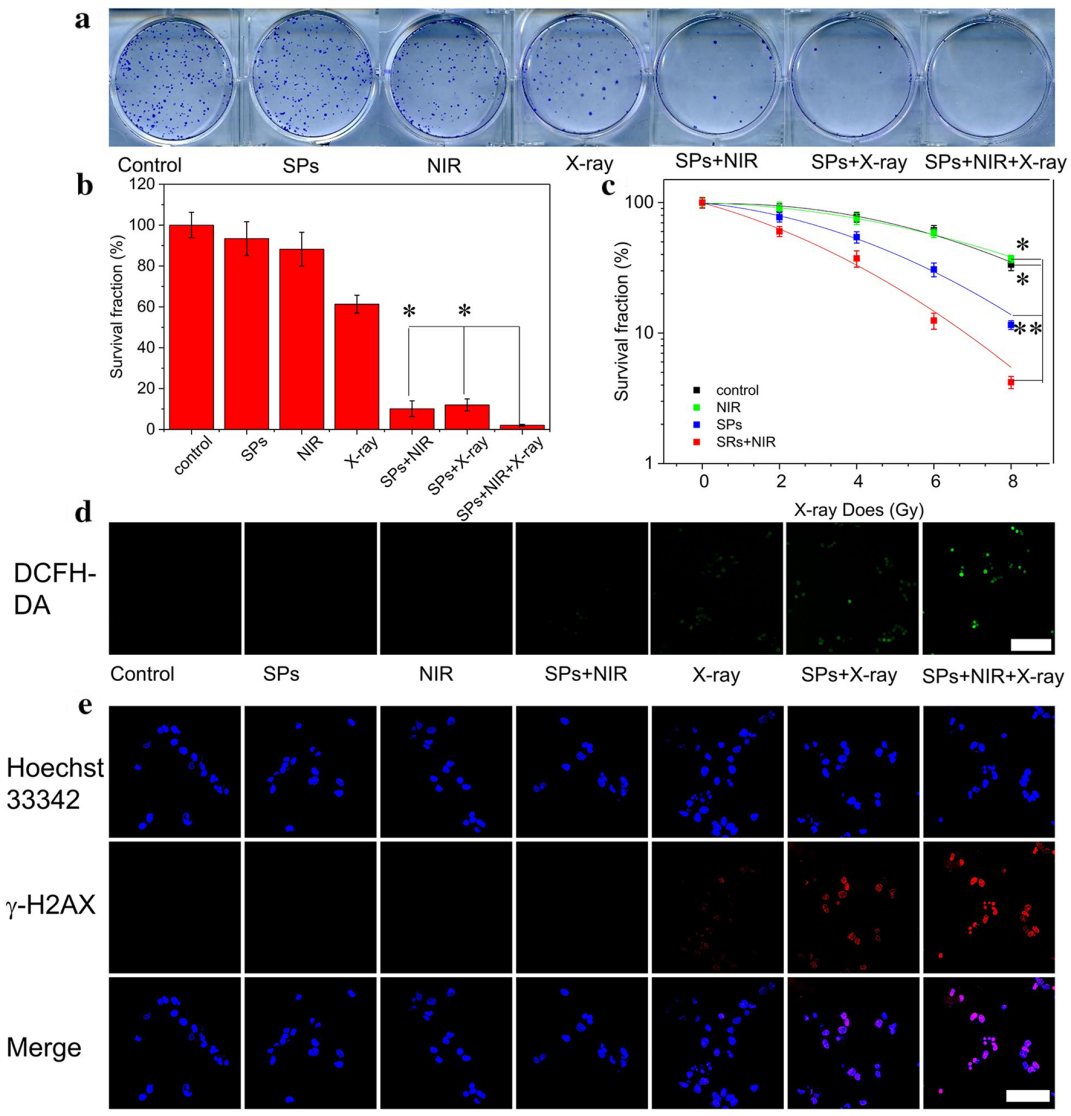
Synergistic cancer treatment properties of BiVO_4_/Fe_3_O_4_@PDA SPs in vitro. **a** Clonogenic assay of KB cells under different treatments (NIR: 0.33 W/cm^2^ and 10 min; X-ray: 6 Gy). **b** Survival fraction of KB cells under different treatments (NIR: 0.33 W/cm^2^ and 10 min; X-ray: 6 Gy). **c** Survival fraction of KB cells under different treatments and X-ray dose. **d** ROS production in KB cells under different treatments (scale bar is 200 µm). **e** γ-H2AX staining in KB cells under different treatments (scale bar is 40 µm). P-values were calculated by one-way ANOVA: *P < 0.05, **P < 0.01

Thereafter, the in vitro synergistic therapeutic effects of BiVO_4_/Fe_3_O_4_@PDA SPs are evaluated via clonogenic assay (Fig. 4a and b). KB cells are treated by X-rays with different radiation doses (2 to 8 Gy) or NIR laser (0.33 W cm^− 2^) in the absence or presence of SPs (100 µg/mL). The result manifests that NIR alone and X-ray alone treatments can decrease the colony formation of KB cells to 88.2% and 61.3%, whereas SPs + NIR and SPs + X-ray can inhibit cell survival to 10.1% and 12.0%. Surprisingly, only 2.1% cells survive after the treatment of SPs + X-ray + NIR. Moreover, compared to the colony forming efficiency under X-ray treatment alone, the same therapeutic effect can be achieved under lower X-ray dose in the X-ray + NIR group, which strongly certify the considerable synergistic therapeutic efficacy between RT and PTT (Fig. 4c).

Next, 2’,7’-dichlorodihydrofluorescein diacetate (DCFH-DA) fluorescent probe is employed to detect intracellular oxidative stress level of KB cells after different treatments [38]. As shown in Fig. 4d, there is no detectable fluorescence in cells treated by PBS, SPs, NIR or SPs + NIR. In contrast, cells under X-ray, SPs + X-ray, SPs + NIR + X-ray treatments exhibit the bright fluorescence, and their fluorescence intensities are gradually enhanced. Since high radiation energy deposition and enhanced oxidative stress may facilitate the damage of DNA, γ-H2AX staining is performed to analyze the damage of DNA double-strand in cell nuclei quantitatively (Fig. 4e) [39]. As similar as the ROS assay, no visible fluorescence is found in cells without the X-ray treatment. The apparent fluorescence can be seen in cells under the treatments of X-ray, SPs + X-ray and SPs + NIR + X-ray, and the SPs + NIR + X-ray treatment produce the highest fluorescence intensity. Both DCFH-DA and γ-H2AX assays demonstrate the synergistic therapeutic efficacy of RT and PTT.

**Fig. 5:**
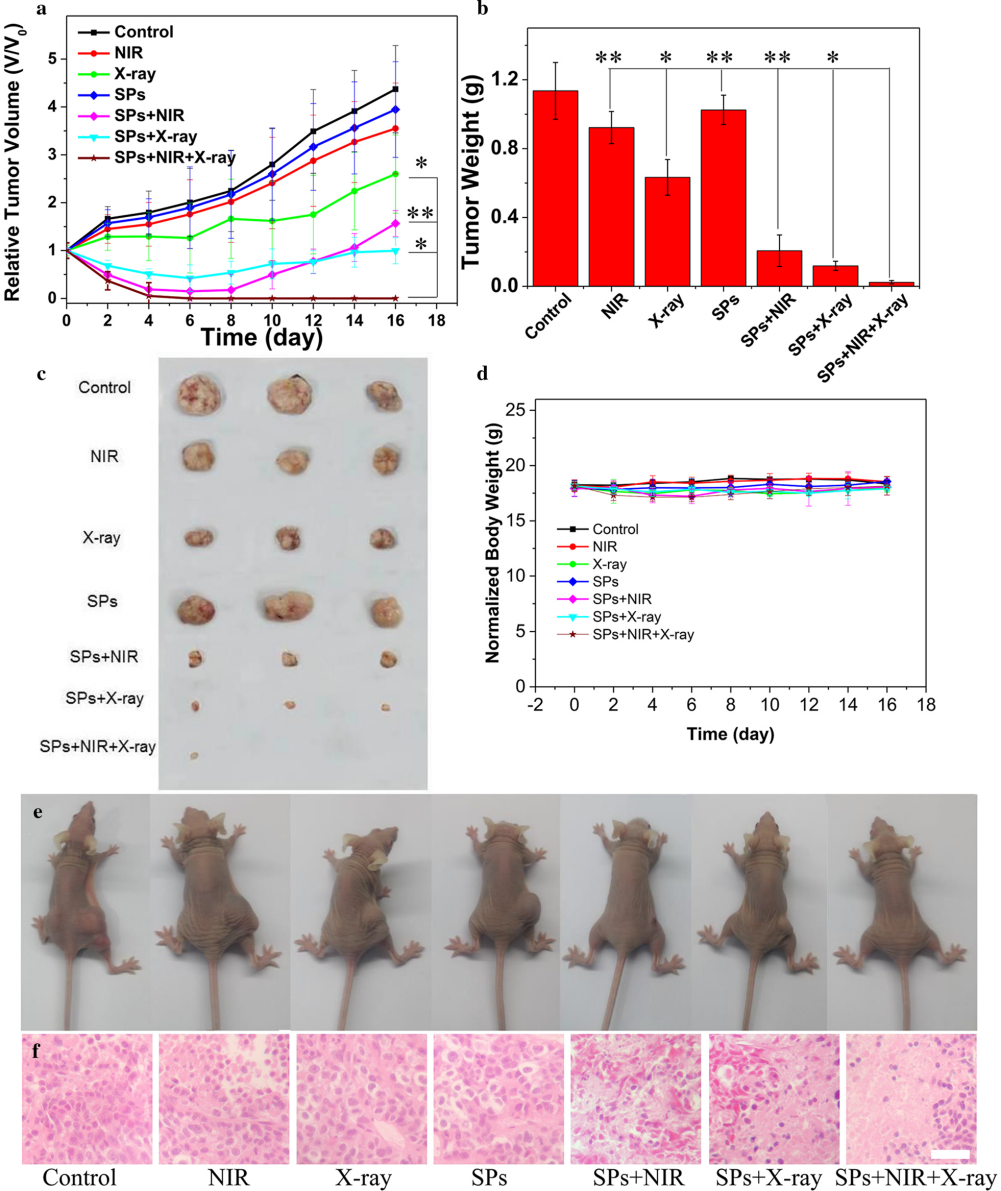
Synergistic cancer treatment properties of BiVO_4_/Fe_3_O_4_@PDA SPs in vivo. Mice are randomly divided into 7 groups with 5 mice in each group. **a** Relative tumor volume curves of mice during 16 d. **b** Average tumor weights at the end of treatment. **c** Tumor photographs at the end of treatment. **d** Body weight curves of mice during 16 d. **e** Photographs of mice at the end of treatment. **f** H&E staining of tumor at the end of treatment (scale bar is 50 µm). P-values were calculated by one-way ANOVA: *P < 0.05, **P < 0.01

Motivated by the effective in vitro therapeutic outcome, the subcutaneous tumor model is employed to investigate the antitumor efficacy of BiVO_4_/Fe_3_O_4_@PDA SPs in vivo. The KB tumor-bearing BALB/c nude mice are randomly divided into 7 groups according to various treatments: (1) PBS, (2) SPs, (3) NIR, (4) X-rays, (5) SPs + NIR, (6) SPs + X-rays, (7) SPs + NIR + X-rays. Mice in group (3), (5) and (7) are irradiated by an 808 nm laser (0.33 W/cm^2^) for 10 min after the injection of BiVO_4_/Fe_3_O_4_@PDA SPs. Additional file 1: Figure S9 exhibits the IR thermographs of mice at different time intervals. The temperature of tumor tissue treated by SPs exhibits a rapid increase of 19°C within 10 min, which is sufficient for tumor ablation. In contrast, there is no significant temperature elevation in the tumor without SPs injection. This dramatic difference lead to the remarkable localized overheat at the tumor site under NIR irradiation, causing severe tumor damage without influencing the adjacent normal tissues. Tumor volumes within 16 days in each group are recorded (Fig. 5a). The tumor volumes in group (1), (2) and (3) increase rapidly, suggesting the negligible effect of SPs alone and NIR alone treatments on the inhibition of tumor growth. Tumors in group (4) grow slowly comparing with those in group (1), revealing the irradiation of X-ray can only inhibit the tumor growth mildly. Despite SPs + X-ray and SPs + NIR treatments have the significantly inhibition on the growth of tumor at the initial stage of treatment, there are recurrences can be found after the treatment for about 10 days. Surprisingly, nearly complete tumor inhibition is realized in group (7) in the absence of recurrence. The weights and photographs of tumors exhibited in Fig. 5b and c further verifies that the synergistic therapeutic efficacy of RT and PTT are better than any single treatment. Because the weights of mice in each group are steady without distinct fluctuation, the side-effects of all the treatments during the therapeutic process can be excluded (Fig. 5e). According to the hematoxylin and eosin (H&E) staining images of tumor tissues (Fig. 5g), the death and nucleus rupture and ablation of cancer cells can be observed in tumors under SPs + X-ray and SPs + NIR treatments, whereas the tumors in group (7) have the most serious cell damage. This result further demonstrates the combination of RT with PTT can greatly improve the therapeutic effect compared to any single treatment. Besides the overlap of RT and PTT, the excellent synergistic therapeutic efficacy of BiVO_4_/Fe_3_O_4_@PDA SPs on tumor inhibition may come from the alleviation of hypoxia status in tumor tissues by boosting intratumoral blood circulation under NIR irradiation. Additional file 1: Figure S10 shows the in vivo PA images of tumors under various treatments: (1) PBS, (2) NIR, (3) SPs, (4) SPs + NIR, which imply that the photothermal conversion capability of BiVO_4_/Fe_3_O_4_@PDA SPs can remarkably increase tumor oxygenation, making tumor cells more sensitive to RT.

At last, the biosafety profile of BiVO_4_/Fe_3_O_4_@PDA SPs is evaluated by using BALB/c nude mice. H&E staining assays of major organs show that different treatments have no significant effects in the organ tissues of heart, liver, spleen, lung and kidney (Additional file 1: Figure S11). Serum biochemistry analysis exhibits the negligible side-effects on blood glucose and lipid, liver and renal function tests after the injection of SPs and combined treatments (Additional file 1: Figure S12). All these results mentioned above testify the excellent biocompatibility and powerful lethality of BiVO_4_/Fe_3_O_4_@PDA SPs.

## Conclusions

In summary, we demonstrate on the design and preparation of BiVO_4_/Fe_3_O_4_@PDA SPs by using BiVO_4_ and Fe_3_O_4_ NPs as the building blocks. BiVO_4_ NPs endow BiVO_4_/Fe_3_O_4_@PDA SPs with impressive X-ray absorption capability due to the high X-ray attenuation coefficient of Bi, which is benefit for their utilization as radiosensitizers for CT imaging and RT. On the other hand, the superparamagnetic of Fe_3_O_4_ NPs strongly guarantee the application of BiVO_4_/Fe_3_O_4_@PDA SPs as T_2_-weighted contrast agent for MR imaging. Furthermore, the aggregation of Fe_3_O_4_ NPs in SPs and the presence of PDA shell greatly improve the photothermal conversion capability of SPs, making BiVO_4_/Fe_3_O_4_@PDA SPs as an ideal photothermal transducer for PA imaging and PTT. By integrating the advantages of various imaging modalities (CT/PA/MR) and therapeutic strategies (RT/PTT), our BiVO_4_/Fe_3_O_4_@PDA SPs exhibit the sensitive multimodal imaging capability and superior synergistic therapeutic efficacy for tumors. Since there are many kinds of building blocks with unique properties appropriating for self-assembly, our work may largely enrich the library of nanomateirals for tumor theranostic.

## Methods

### Materials

Bi(NO_3_)_3_·5H_2_O (99.0%, Aladdin), NH_4_VO_3_ (99.9%, Aladdin), Fe(acac)_3_ (99.9 %, Simga-Aldrich), oleyamine (OLA, 70%, Simga-Aldrich), 1,2-hexadecanediol (90%, Aladdin), oleic acid (OA, 90 %, Simga-Aldrich), SDS (99%, Simga-Aldrich), 1-octadecene (ODE, 90 %, Simga-Aldrich), tris(hydroxymethyl) aminomethans (Tris, > 99%, Aladdin), DA (99.0%). γ-H2AX (phospho S139) antibody [EP854(2)Y] (Alexa Fluor 568) (ab206901) was purchased from Abcam. Crystal violet staining solution, CCK-8, hoechst 33,342 and ROS assay kit were purchased from Beyotime.

### Preparation of BiVO_4_ NPs

1 mmol Bi(NO_3_)_2_·5H_2_O, 2 mL OLA, 2 mL OA and 10 mL ODE were added into a 100 mL three-necked flask. The temperature was raised to 175 °C in nitrogen atmosphere under vigorous stirring. When the solution was completely clear, the solution was dropped to 130 °C, followed by the addition of 10 mL water containing 2 mmol NH_4_VO_3_. The resulting solution was heated at 100  °C for 5 min and cooled down to room temperature naturally. After that, the solution was uniformly mixed with ethanol, and the bottom aqueous layer of the mixture was discarded. The upper organic layer was mixed with water and ethanol for twice, and the bottom aqueous layer of the mixture was discarded. The final product was washed by ethanol for another three times and dispersed in toluene.

### Preparation of Fe_3_O_4_ NPs

OA-capped Fe_3_O_4_ NPs were prepared by the thermal decomposition route. 2 mmol Fe(acac)_3_, 2 mL OA, 2 mL OLA, 5 mmol 1,2-hexadecanediol and 20 mL benzyl ether were added into a 100 mL three-necked flask. The mixture was heated at 200 °C for 30 min under nitrogen atmosphere, followed by reflux at 265 °C for 30 min. After that, the solution was dropped to room temperature naturally, and the product was washed for three times by ethanol and finally dispersed in toluene.

### Preparation of SDS-capped BiVO_4_/Fe_3_O_4_ SPs

2 mL toluene containing 50 mg BiVO_4_ NPs and 20 mg Fe_3_O_4_ NPs was added into 5 mL aqueous solution containing 10 mg SDS. After ultrasonic stirring for 10 min, the resulting emulsions were heated at 60 °C for another 30 min to evaporate toluene. After that, solution was centrifuge at 3000 r/min for 5 min, and the SDS-capped BiVO_4_/Fe_3_O_4_ SPs were obtained.

### Preparation of BiVO_4_/Fe_3_O_4_@PDA SPs

DA monomer was added into 10 mL Tris-buffer solution (10 mM, pH 8.5) containing 5 mg SDS-capped BiVO_4_/Fe_3_O_4_ SPs. After stirring for 3 h, the reaction solution was centrifuged at 5000 r/min for 30 min. Then, BiVO_4_/Fe_3_O_4_@PDA SPs were obtained.

### Characterization

TEM was taken on a Hitachi H-800 electron microscope (200 kV) coupled with a CCD camera. UV-vis absorption spectra were obtained using a Lambda 800 UV-vis spectrophotometer. HRTEM and EDS were performed on a JEM-2100F electron microscope at an acceleration voltage of 200 kV with an EDS detector. XRD was implemented on an Empyrean X-ray diffractometer with Cu K radiation (λ = 1.5418 Å).

### Cell experiment

#### Cytotoxicity assay

We used CCK-8 and KB cells to test the cytotoxicity of SPs. In the 96-well plate, we cultured 5000 cells for each well. The cells were incubated at 37 °C with 5% CO_2_ for 24 h. Then the cells were treated with different concentrations of SPs and further incubated for 24 h. After that, the medium was removed and the cells were washed twice with PBS. Solarbio 1640 medium is re-added with 10 µL of CCK-8 and the cells were continued to culture for 1 h. Finally, the microplate reader was used to measure the absorbance at 450 nm.

### Clonogenic assay

In the 6-well plate, we cultured 1000 KB cells for each well. After co-cultivation with SPs (100 µg/mL) overnight, the cells were first treated with NIR (0.33 W/cm^2^, 10 min), then X-rays (0, 2, 4, 6, 8 Gy) sequentially. After that, the cells were washed with PBS and continued to incubate for 10 days. Finally, the cells were stained with crystal violet staining solution. Surviving fraction (SF) was calculated by (surviving colonies)/(cells seeded × plating efficiency). The mean surviving fraction was obtained from three parallel samples. The SF and the radiation dose can be fitted using the following formula: SF = exp[-(αD + βD^2^)]

### ROS in cells

We use ROS assay kit to detect ROS in cells. In the 6-well plate, we cultured 50,000 KB cells in each well. We used SPs (100 µg/mL) to co-cultivate with cells overnight, followed by washing the cells with PBS for three times. We added the 1000-fold diluted ROS assay kit to each well and washed the cells with PBS for three times after incubation for 20 min. Then we treated the cells with X-ray (6 Gy) or NIR (0.33 W/cm^2^, 10 min). The cells were first irradiated by NIR, then by X-ray. Finally, the cells were observed using the FV1000 laser scanning confocal microscopy (excitation wavelength: 488 nm; emission wavelength: 525 nm).

###  DNA double‐strand breaks

In the 6-well plate, we cultured 50,000 KB cells in each well. We used SPs (100 µg/mL) to co-cultivate with cells overnight, followed by treatment of cells with X-ray (6 Gy) or NIR (0.33 W/cm^2^, 10 min). The cells were first irradiated by NIR, then by X-ray. We fixed the cells with paraformaldehyde (4%) for 10 min and washed with PBS for three times. It was then permeabilized with methanol and washed with PBS for three times. The cells were exposed in blocking buffer for 1 h and further incubated with 100-fold γ-H2AX (phospho S139) antibody [EP854(2)Y] (Alexa Fluor 568) (ab206901) at 4 °C overnight and then washed with PBS for three times (excitation wavelength: 578 nm; emission wavelength: 603 nm). The cells were stained with Hoechst 33,342 (excitation wavelength: 350 nm; emission wavelength: 461 nm) and washed with PBS for three times. Finally, the cells were observed using FV1000 laser scanning confocal microscopy.

### Animal experiment

#### ***In vivo*** RT/PTT synergistic treatment

KB cells were subcutaneously injected into the right leg of BALB/c nude mice. When the average tumor volume reached 75 mm^2^, mice were randomly divided into 7 groups with 5 mice in each group according to different treatment conditions: (1) PBS, (2) SPs, (3) NIR, (4) X-rays, (5) SPs + NIR, (6) SPs + X-rays, (7) SPs + NIR + X-rays. We injected 20 µL of SPs (5 mg/mL) into the tumor via intratumoral injection. Mice in the corresponding groups were then treated with NIR (0.33 W/cm^2^, 10 min) or X-ray (6 Gy). Tumor volumes and body weights of mice in each group were recorded after this treatment. Finally, all the mice were sacrificed and the blood, heart, liver, spleen, lung, kidney and tumor were taken. The blood was centrifuged and the serum was taken for the blood analysis. The tumors and other organs were weighed and used for H&E staining.

#### CT imaging

In vivo and in vitro CT imaging were used U-SPECT+/CT (MILABS). For in vitro imaging, we prepared aqueous solutions of different concentrations of SPs and iopromide and measured their HU values. For in vivo imaging, BALB/c nude mice harboring KB tumors were intratumoral injected with 50 µL, 10 mg/mL SPs solution.

#### MR imaging

We used SIEMENS Avanto 1.5T clinical MRI unit to assess the MR imaging property of SPs. For in vitro imaging, we prepared aqueous solutions of different concentrations of SPs for measuring. For in vivo imaging, BALB/c nude mice with KB tumors were intratumoral injected with 20 µL, 10 mg/mL SPs solution.

#### PA imaging

In vivo and in vitro PA imaging was used MSOT INVISIO-256 (iThera Medical). For in vitro imaging, we prepared aqueous solutions of different concentrations of SPs for measuring. For in vivo imaging, BALB/c nude mice with KB tumors were intratumoral injected with 20 µL, 10 mg/mL SPs solution.

## Supplementary Information

**Additional file 1: Figure S1.** XRD patterns of BiVO_4_ and Fe_3_O_4_ NPs. **Figure S2.** EDS-Mappnig images of the BiVO_4_/Fe_3_O_4_ SPs. **Figure S3.** TEM images of BiVO_4_/Fe_3_O_4_@PDA SPs with different sizes. **Figure S4.** TEM images of BiVO_4_/Fe_3_O_4_@PDA SPs with different thicknesses of the PDA shell. **Figure S5.** UV-vis absorption spectra of BiVO_4_ NPs, Fe_3_O_4_ NPs, BiVO_4_/Fe_3_O_4_ SPs and BiVO_4_/Fe_3_O_4_@PDA SPs. **Figure S6.** Photothermal conversion efficiency calculation of BiVO_4_/Fe_3_O_4_@PDA SPs. **Figure S7.** Concentration-related cytotoxicity of BiVO_4_/Fe_3_O_4_@PDA SPs. **Figure S8.** Colloidal stability of BiVO_4_/Fe_3_O_4_@PDA SPs. **Figure S9.** Infrared imaging photographs of KB tumor-bearing mice with or without SPs injection. **Figure S10.** PA imaging of tumor oxygenation of tumors under the treatments. **Figure S11.** H&E stained photographs of organs after treatment. **Figure S12.** Blood biochemistry analyses of mice after different treatments. **Table. S1.** BiVO_4_/Fe_3_O_4_ SPs with different Bi/Fe element ratios.

## Data Availability

All sequence data generated and analysed during the current study are available in the NCBI database under the project accession number PRJNA597946, (https://www.ncbi.nlm.nih.gov/sra/PRJNA597946).
