# Supplementary material for: BiVO4/Fe3O4@polydopamine superparticles for tumor multimodal imaging and synergistic therapy
Source: J Nanobiotechnology. 2021 Mar 29;19:90. doi: 10.1186/s12951-021-00802-x (PMC8008624; doi:10.1186/s12951-021-00802-x)
Supplement: Supplementary file 1 — Additional file 1: Figure S1. XRD patterns of BiVO4 and Fe3O4 NPs. Figure S2. EDS-Mappnig images of the BiVO4/Fe3O4 SPs. Figure S3. TEM images of BiVO4/Fe3O4@PDA SPs with different sizes. Figure S4. TEM images of BiVO4/Fe3O4@PDA SPs with different thicknesses of the PDA shell. Figure S5. UV-vis absorption spectra of BiVO4 NPs, Fe3O4 NPs, BiVO4/Fe3O4 SPs and BiVO4/Fe3O4@PDA SPs. Figure S6. Photothermal conversion efficiency calculation of BiVO4/Fe3O4@PDA SPs. Figure S7. Concentration-related cytotoxicity of BiVO4/Fe3O4@PDA SPs. Figure S8. Colloidal stability of BiVO4/Fe3O4@PDA SPs. Figure S9. Infrared imaging photographs of KB tumor-bearing mice with or without SPs injection. Figure S10. PA imaging of tumor oxygenation of tumors under the treatments. Figure S11. H&E stained photographs of organs after treatment. Figure S12. Blood biochemistry analyses of mice after different treatments. Table. S1. BiVO4/Fe3O4 SPs with different Bi/Fe element ratios. [file 12951_2021_802_MOESM1_ESM.docx]

BiVO_4_/Fe_3_O_4_@Polydopamine Superparticles for Tumor Multimodal Imaging and Synergistic Therapy

Ze Wang,^a,#^ Guan Wang,^d,#^ Tingting Kang,^a,#^ Shuwei Liu,^a^ Lu Wang,^e^ Haoyang Zou,^b,^* Yu Chong^c,^* and Yi Liu^a,^*

*^a^State Key Laboratory of Supramolecular Structure and Materials, Jilin University, Changchun 130012, P. R. China. *Address correspondence to yiliuchem@jlu.edu.cn*

*^b^Key Laboratory of Polymer Ecomaterials, Changchun Institute of Applied Chemistry, Chinese Academy of Sciences, Changchun 130012, P. R. China.*

*^c^State Key Laboratory of Radiation Medicine and Protection, School for Radiological and Interdisciplinary Sciences (RAD-X), Collaborative Innovation Center of Radiation Medicine of Jiangsu Higher Education Institutions, Soochow University, Suzhou, 215123, P. R. China.*

*^d^Department of Gastroenterology, China-Japan Union Hospital, Jilin University, Changchun 130033, P. R. China.*

*^e^Department of Oral Pathology, School and Hospital of Stomatology, Jilin University, Changchun 130021, P. R. China.*

*^#^These authors contributed equally to this work.*

**Table of the Electronic Supplementary Information**

**1.** **Fig. S1** XRD patterns of BiVO_4_ and Fe_3_O_4_ NPs.

**2.** **Fig. S2** EDS-Mappnig images of the BiVO_4_/Fe_3_O_4_ SPs.

**3.** **Fig. S3** TEM images of BiVO_4_/Fe_3_O_4_@PDA SPs with different sizes.

**4.** **Fig. S4** TEM images of BiVO_4_/Fe_3_O_4_@PDA SPs with different thicknesses of the PDA shell.

**5.** **Fig. S5** UV-vis absorption spectra of BiVO_4_ NPs, Fe_3_O_4_ NPs, BiVO_4_/Fe_3_O_4_ SPs and BiVO_4_/Fe_3_O_4_@PDA SPs.

**6.** **Fig. S6** Photothermal conversion efficiency calculation of BiVO_4_/Fe_3_O_4_@PDA SPs.

**7. Fig. S7** Concentration-related cytotoxicity of BiVO_4_/Fe_3_O_4_@PDA SPs.

**8. Fig. S8** Colloidal stability of BiVO_4_/Fe_3_O_4_@PDA SPs.

**9. Fig. S9** Infrared imaging photographs of KB tumor-bearing mice with or without SPs injection.

**10. Fig. S10** PA imaging of tumor oxygenation of tumors under the treatments.

**11. Fig. S11** H&E stained photographs of organs after treatment.

**12. Fig. S12** Blood biochemistry analyses of mice after different treatments.

**13. Table. S1** BiVO_4_/Fe_3_O_4_ SPs with different Bi/Fe element ratios.

**Fig.S1** XRD patterns of BiVO_4_ and Fe_3_O_4_ NPs.


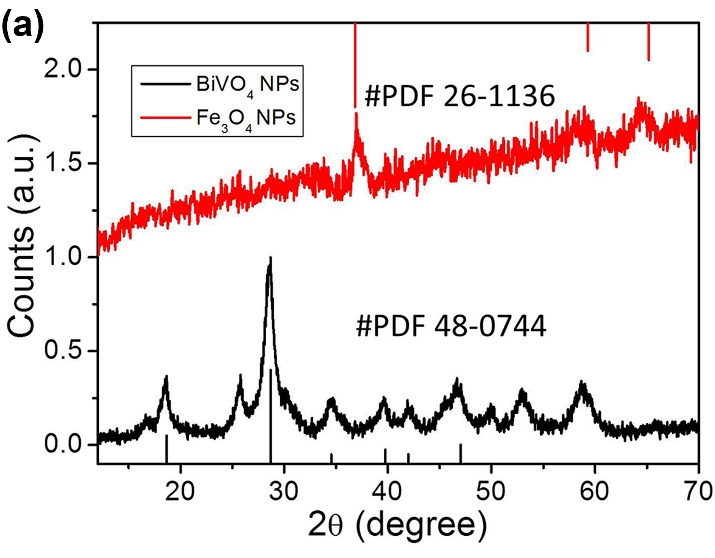


**Fig. S2** EDS-Mappnig images of the BiVO_4_/Fe_3_O_4_ SPs.

**
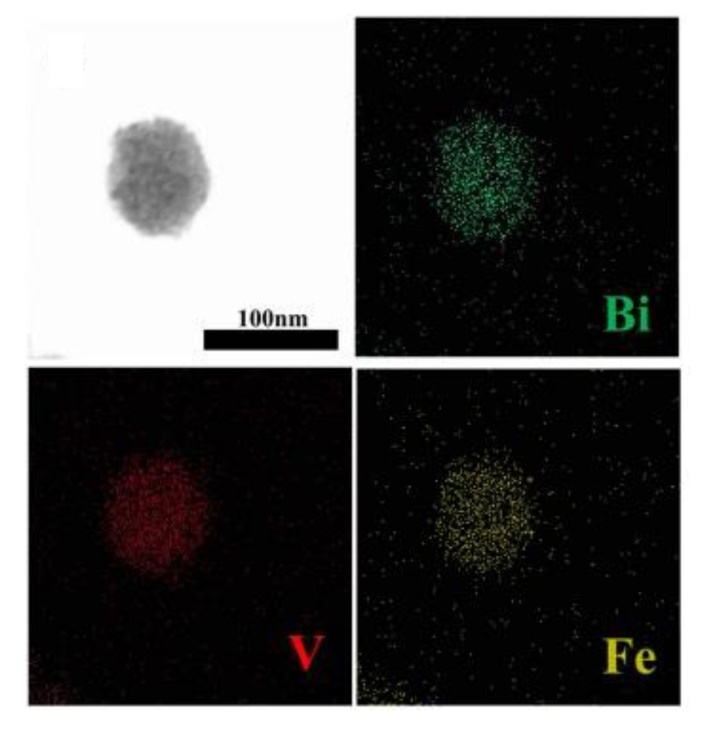
**

**Fig.S3** TEM images of BiVO_4_/Fe_3_O_4_@PDA SPs with the sizes of 81.20 nm (a), 120.60 nm (b), and 164.50 nm (c).


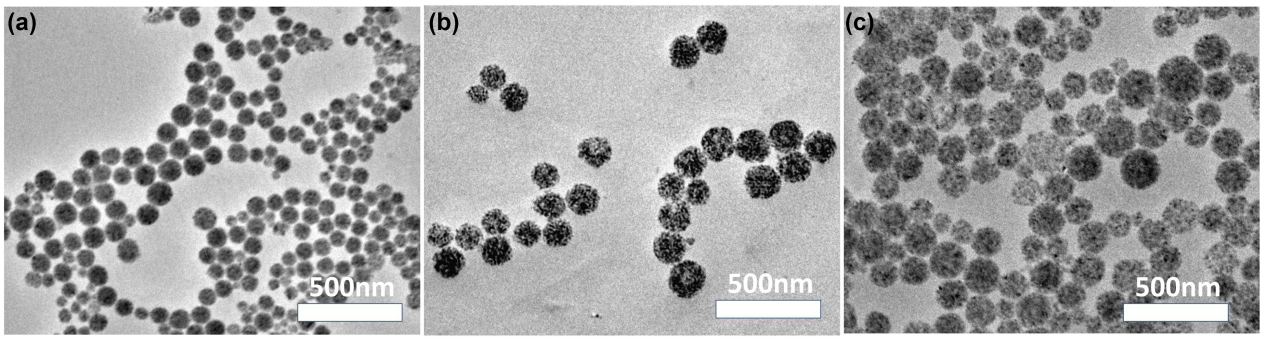


**Fig. S4** TEM images of BiVO_4_/Fe_3_O_4_@PDA SPs with the PDA shell thicknesses from 10.00 to 80.00 nm.


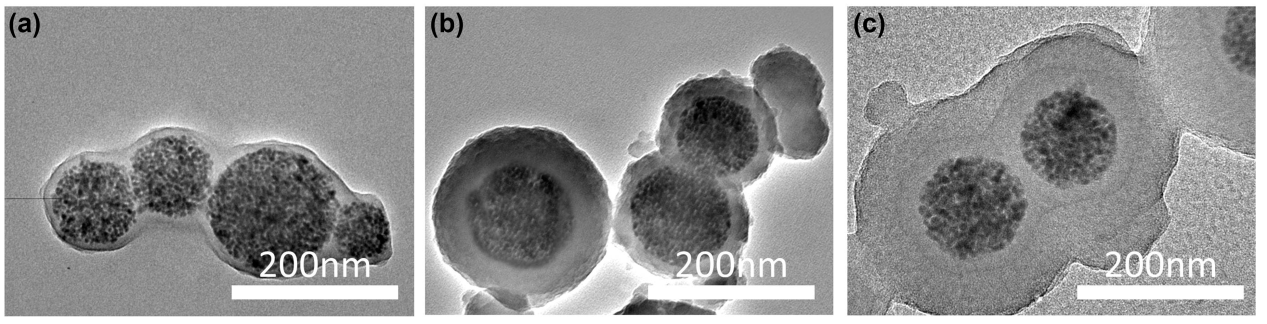


**Fig. S5** UV-vis absorption spectra of BiVO_4_ NPs, Fe_3_O_4_ NPs, BiVO_4_/Fe_3_O_4_ SPs and BiVO_4_/Fe_3_O_4_@PDA SPs.


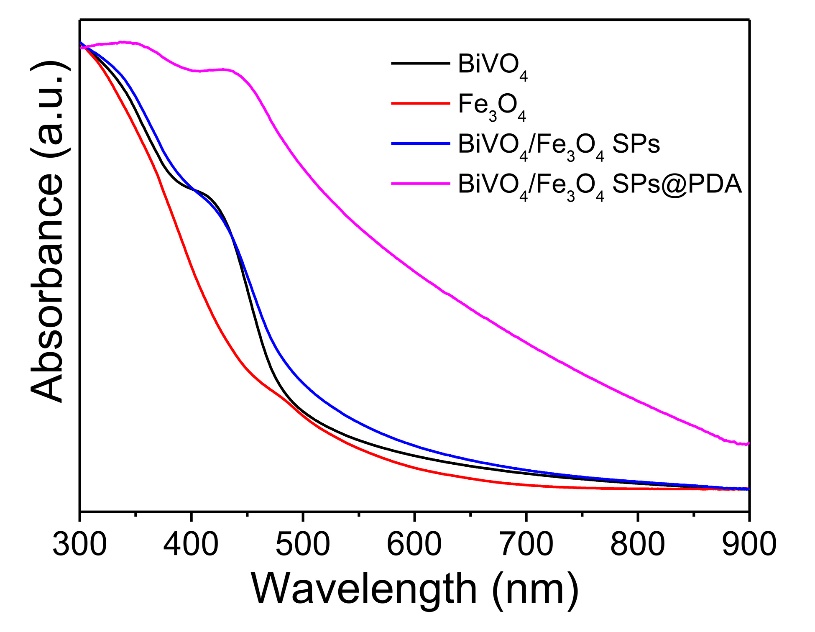


**Fig. S6** (a) Temperature rises and fall curve of 100 μg/mL BiVO_4_/Fe_3_O_4_-2@PDA SPs under 1 W/cm^2^ 808 nm laser irradiation. (b) t=-τlnθ relationship diagram calculated by (a). The photothermal conversion efficiency was calculated to be 33.42%.


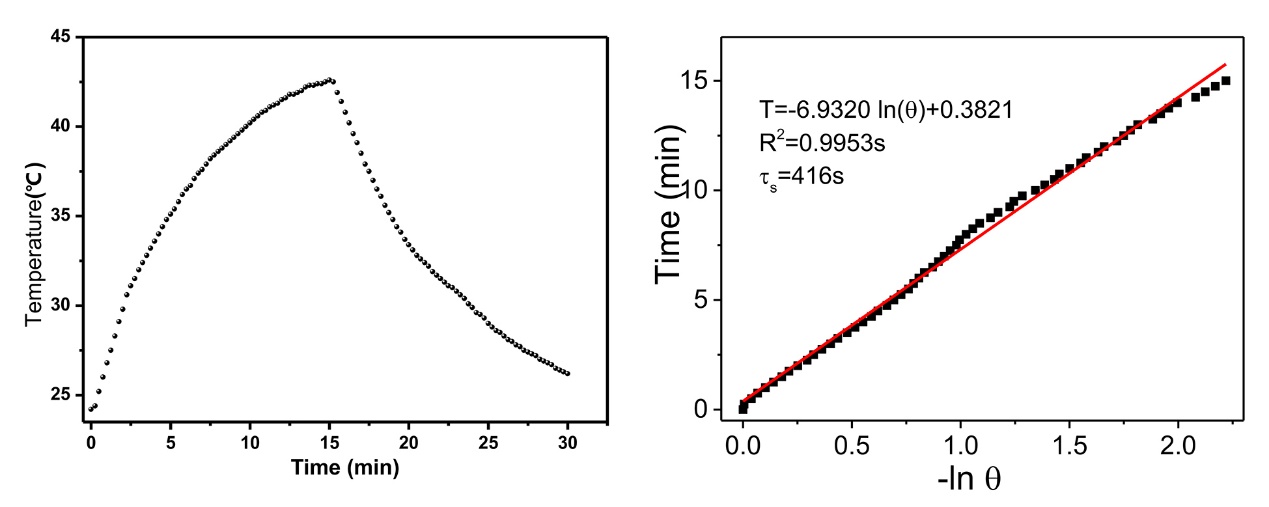


**Fig. S7** Cell viability of KB cells after incubation with various concentrations of BiVO_4_/Fe_3_O_4_@PDA SPs for 24 h.


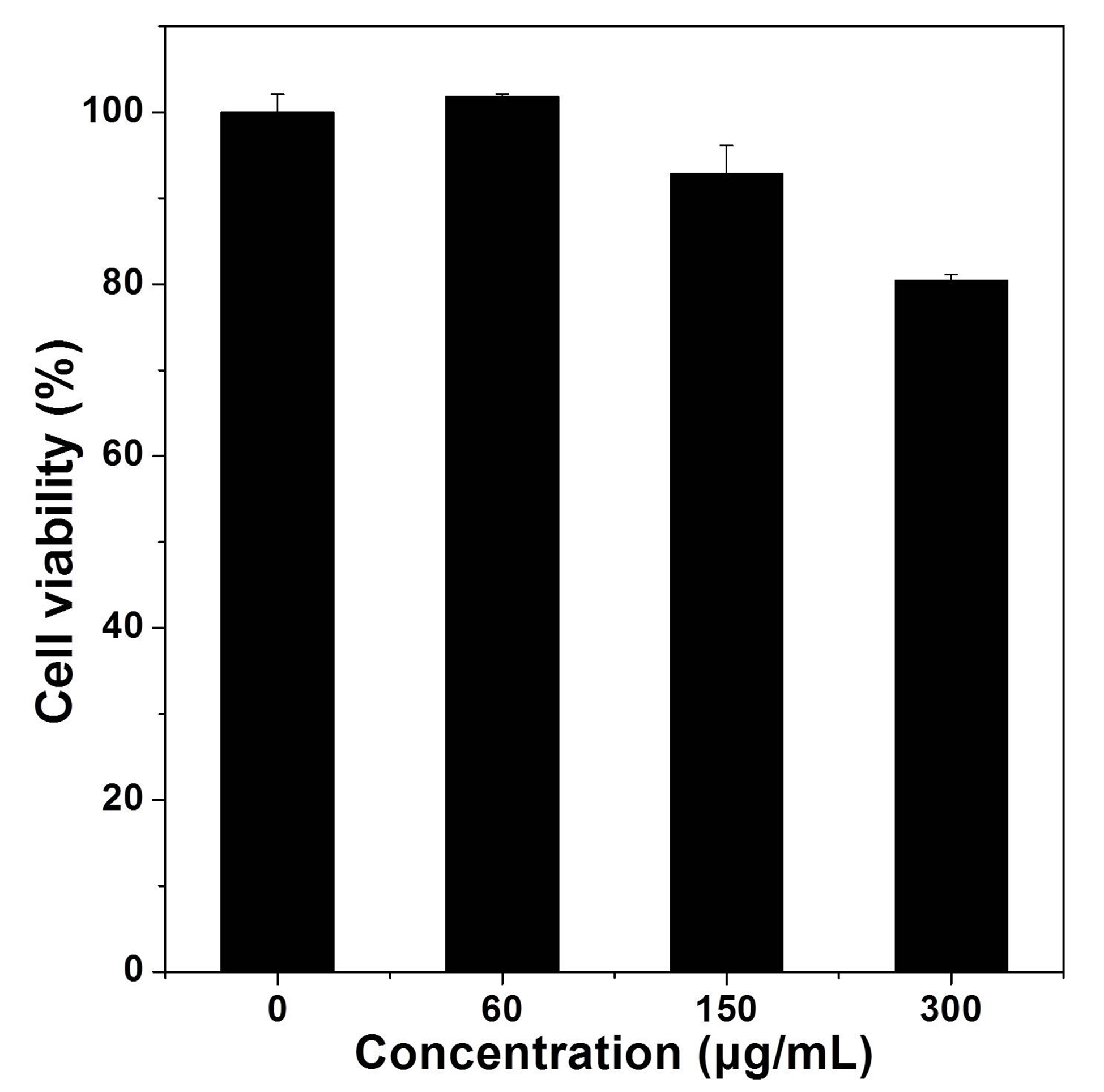


**Fig. S8** Photographs of BiVO_4_/Fe_3_O_4_@PDA SPs before and after storage in deionized water, saline, PBS, cell culture and serum-containing cell culture for 24h.


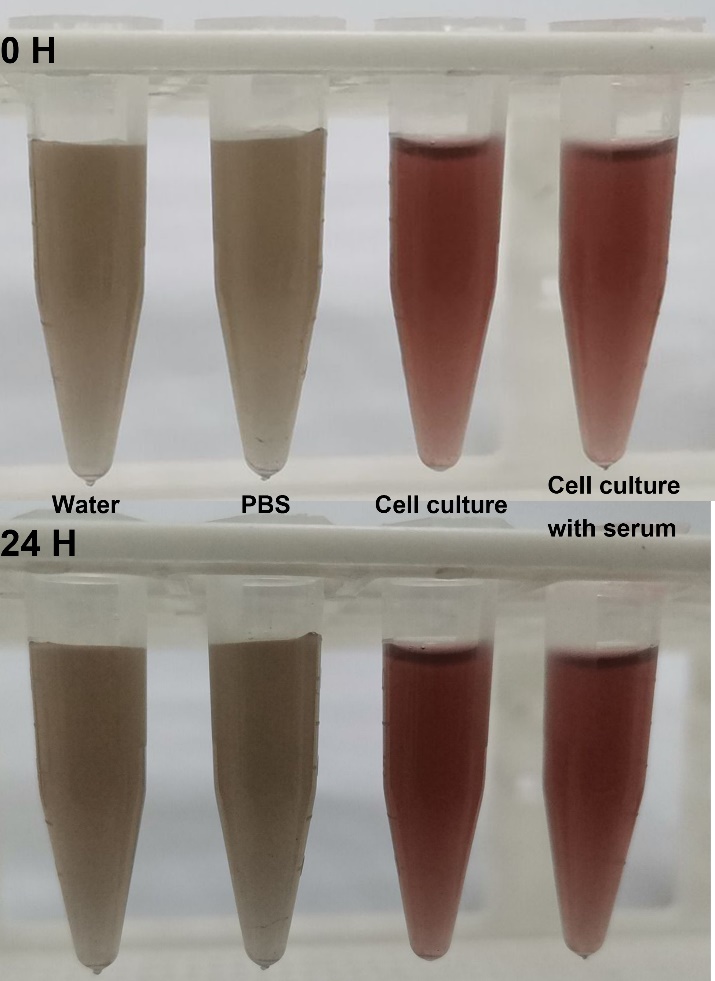


**Fig. S9** Infrared imaging photographs of KB tumor-bearing mice with or without SPs injection (laser, 808 nm; 0.33 W/cm^2^).

**
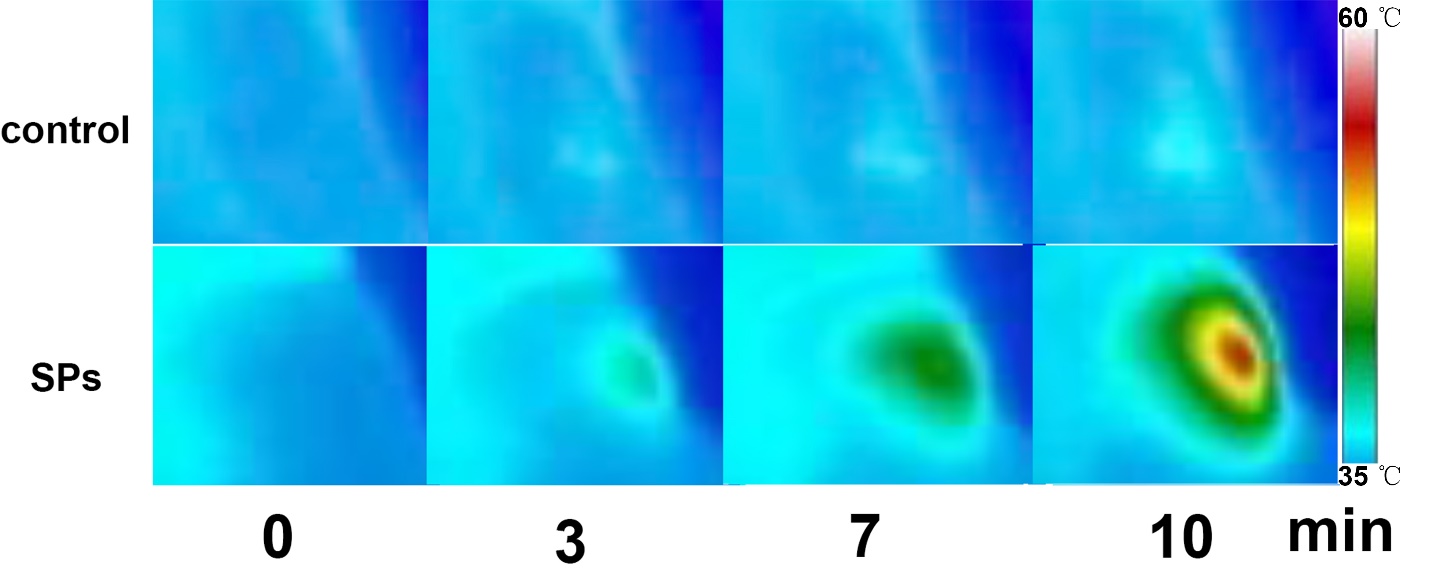
**

**Fig. S10** PA imaging of tumor oxygenation of tumors under the treatments by PBS (a), NIR (b), SPs (c) and SPs+NIR (d). Oxygenated hemoglobin is colored red, and deoxygenated hemoglobin is colored blue.


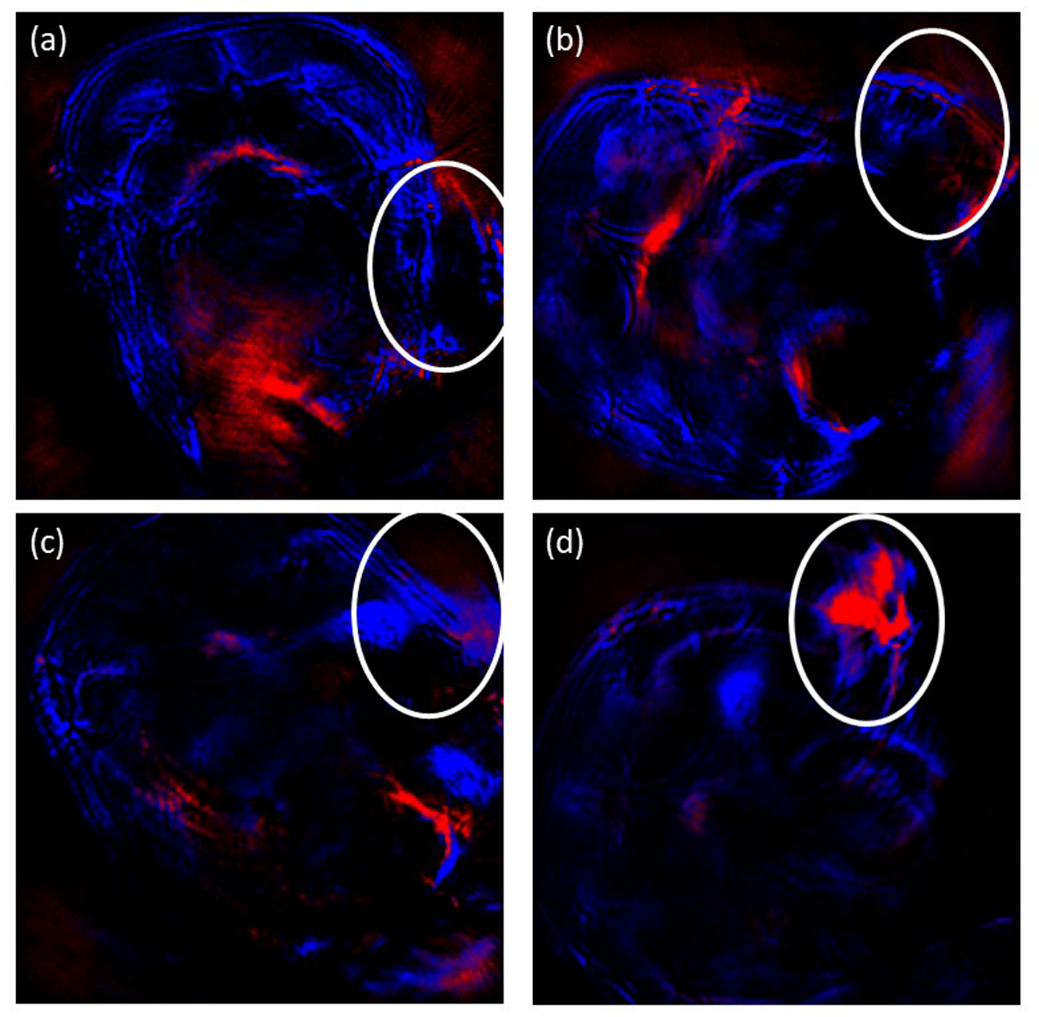


**Fig. S11** H&E stained photographs of organs (heart, liver, spleen, lung, kidney) of mice in each group after treatment (scale bar is 50 μm).


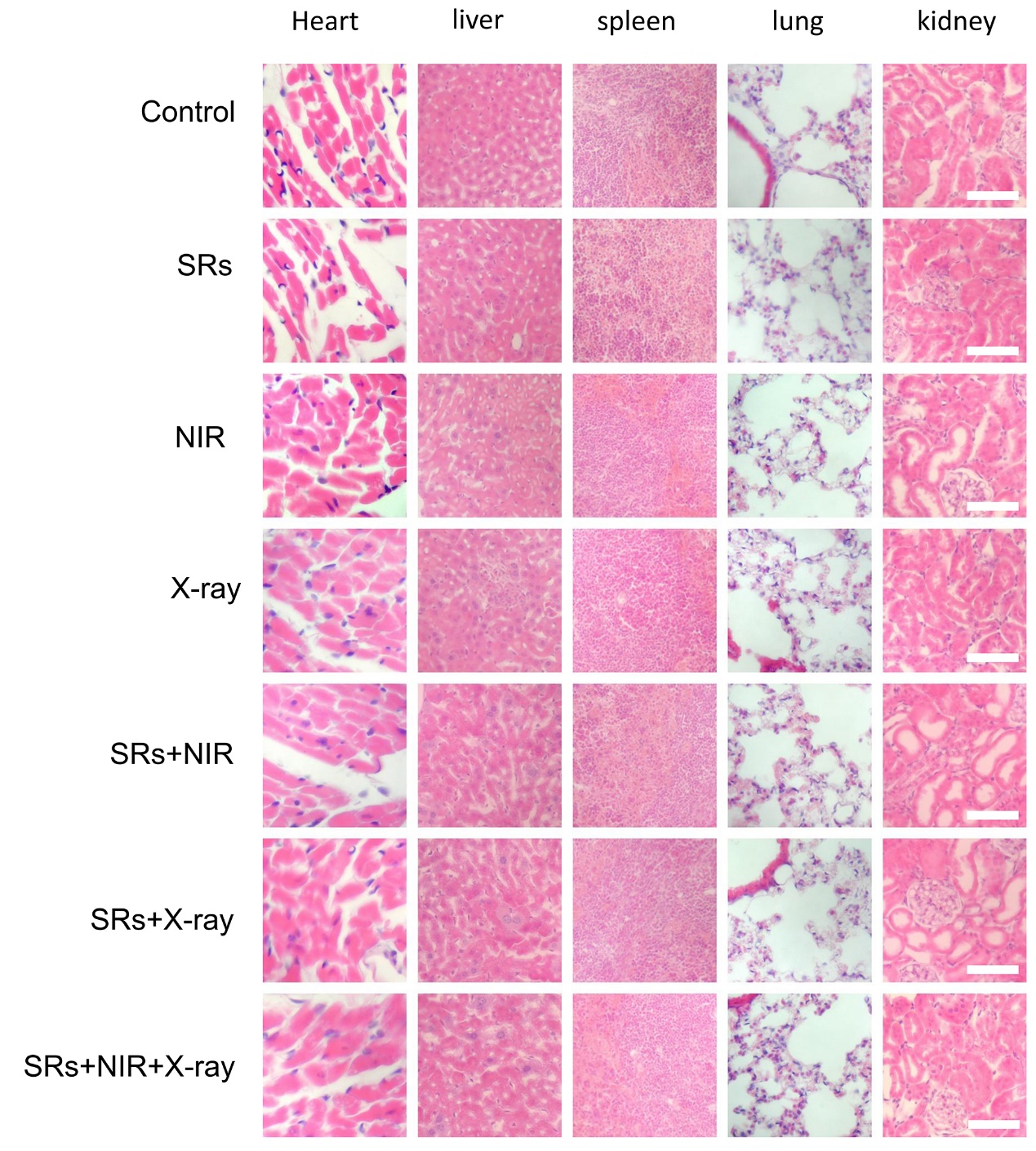


**Fig. S12** Blood biochemistry analyses of mice after different treatments.


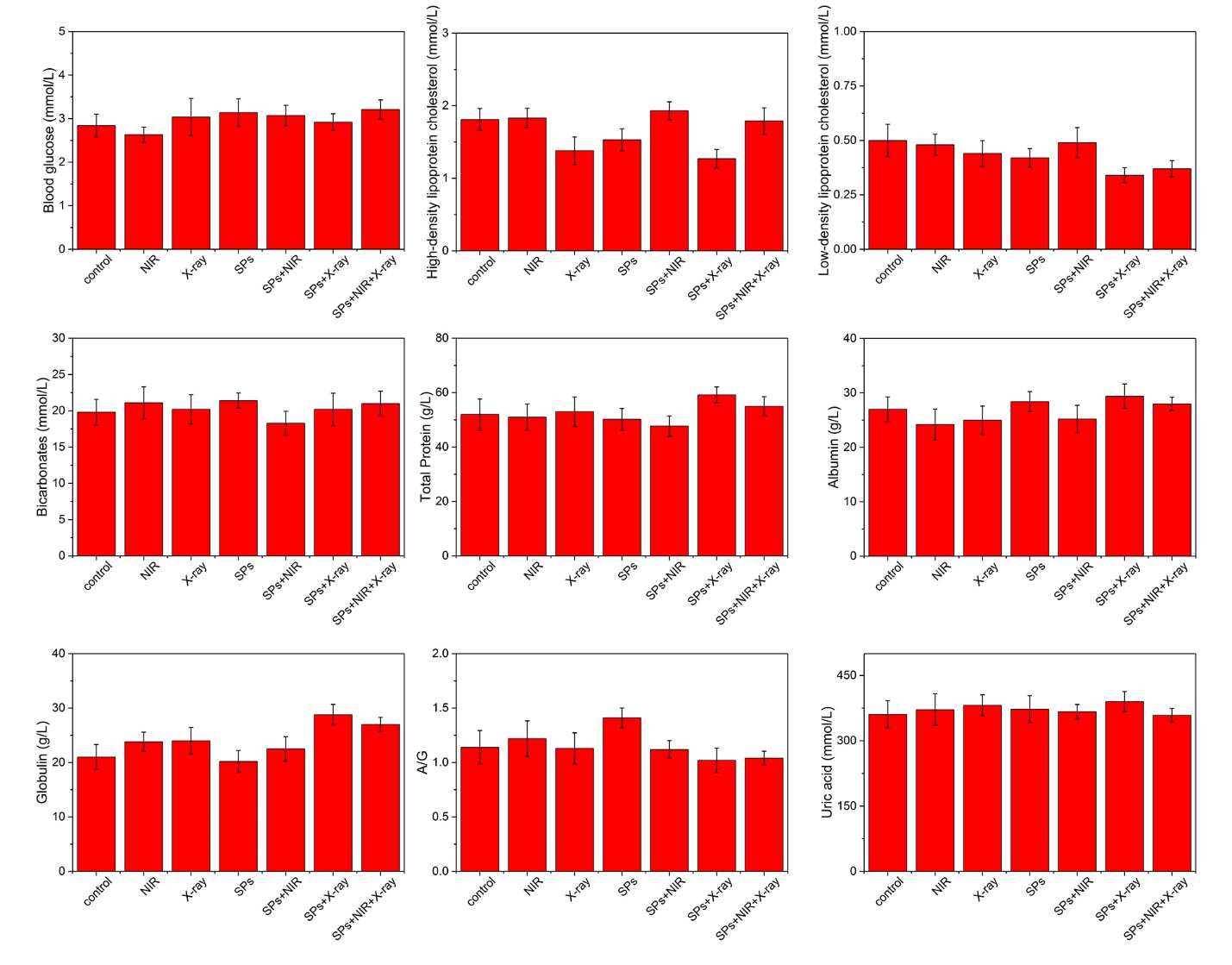


**Table S1** BiVO_4_/Fe_3_O_4_ SPs with different Bi/Fe element ratios.

| Samples | BiVO_4_/Fe_3_O_4_-1 SPs | BiVO_4_/Fe_3_O_4_-2 SPs | BiVO_4_/Fe_3_O_4_-3 SPs |
| --- | --- | --- | --- |
| Bi:Fe | 3.5:1 | 1.8:1 | 1.2:1 |
